# Urine-derived cells: a promising diagnostic tool in Fabry disease patients

**DOI:** 10.1038/s41598-018-29240-w

**Published:** 2018-07-23

**Authors:** Gisela G. Slaats, Fabian Braun, Martin Hoehne, Laura E. Frech, Linda Blomberg, Thomas Benzing, Bernhard Schermer, Markus M. Rinschen, Christine E. Kurschat

**Affiliations:** 1 Department II of Internal Medicine Medicine and Center for Rare Diseases Cologne, University Hospital of Cologne, Cologne, Germany; 2 Cologne Excellence Cluster on Cellular Stress Responses in Ageing-Associated Diseases (CECAD), University of Cologne, Cologne, Germany; 3 Center for Molecular Medicine Cologne, University of Cologne, Cologne, Germany; 4 III. Department of Medicine, University Medical Center Hamburg-Eppendorf, Hamburg, Germany; 5 Systems Biology of Aging, University of Cologne, Cologne, Germany

## Abstract

Fabry disease is a lysosomal storage disorder resulting from impaired alpha-galactosidase A (α-Gal A) enzyme activity due to mutations in the *GLA* gene. Currently, powerful diagnostic tools and *in vivo* research models to study Fabry disease are missing, which is a major obstacle for further improvements in diagnosis and therapy. Here, we explore the utility of urine-derived primary cells of Fabry disease patients. Viable cells were isolated and cultured from fresh urine void. The obtained cell culture, modeling the renal epithelium, is characterized by patient-specific information. We demonstrate that this non-invasive source of patient cells provides an adequate cellular *in vivo* model as cells exhibit decreased α-Gal A enzyme activity and concomitant globotriaosylceramide accumulation. Subsequent quantitative proteomic analyses revealed dysregulation of endosomal and lysosomal proteins indicating an involvement of the Coordinated Lysosomal Expression and Regulation (CLEAR) network in the disease pathology. This proteomic pattern resembled data from our previously described human podocyte model of Fabry disease. Taken together, the employment of urine-derived primary cells of Fabry disease patients might have diagnostic and prognostic implications in the future. Our findings pave the way towards a more detailed understanding of pathophysiological mechanisms and may allow the development of future tailored therapeutic strategies.

## Introduction

Fabry disease [MIM: 301500] is a hereditary disorder of the glycosphingolipid metabolism caused by mutations in the alpha-galactosidase A (*GLA*) gene located on the X chromosome. Male patients mostly present with more severe symptoms of the disorder. Due to impaired activity or total loss of the enzyme alpha-galactosidase A (α-Gal A), substrates with terminally α-glycosidically bound galactose, primarily globotriaosylceramide (Gb3), are accumulating in enlarged lysosomes of cells within virtually every organ and tissue in the affected individuals. Consistently, patients display a wide spectrum of symptoms and disorders such as severe neuropathic pain, ischemic stroke at very young age, cardiomyopathy, renal insufficiency, gastrointestinal dysfunction, heat sensitivity, hypohidrosis and fever^1^. Cardiologic complications and end-stage renal disease (ESRD) are primarily responsible for premature death in these patients. Over the course of the disease, patients develop progressive Gb3 depositions, most prominent in podocytes, with following glomerulosclerosis and interstitial fibrosis with tubular atrophy^1,2^. ESRD is frequently preceded by (micro-) albuminuria and can coincide with the development of hypertension. A plethora of unspecific symptoms in Fabry patients contributes to the fact that most individuals are diagnosed late in the course of their disease. To date, research has focused on the development of new biomarkers for diagnosis but also for monitoring disease course and treatment efficacy. Nevertheless, the portfolio of routine biomarkers and disease models is still very limited. For these reasons, we propose to introduce primary urine cells of Fabry disease patients as a diagnostic tool.

Fabry disease is treated either with intravenous enzyme replacement or with oral chaperone therapy^3,4^. Fabry therapy ameliorates disease progression and clinical symptoms. Recent studies have suggested that lyso-Gb3, the Gb3 derivative globotriaosylsphingosine, is a potential tool for monitoring treatment efficacy in Fabry disease patients^5,6^. To date, additional biomarkers for monitoring disease progression are lacking^7^.

In contrast to blood samples or skin biopsies, urine-derived cell cultures have been shown to be a non-invasive important source of cells which – despite their heterogeneity – mirror pathomechanisms in hereditary diseases affecting the kidney^8–10^. Here, we describe the use of urine-derived primary cells of Fabry disease patients as an effective tool for diagnosis and research of pathomechanisms. In cultured urine-derived primary cells of Fabry disease patients we detected decreased α-Gal A enzyme activity and concomitant Gb3 accumulation. Furthermore, quantitative proteomic analyses confirmed the loss of α-Gal A protein and revealed dysregulation of lysosomal proteins. For future studies on disease mechanisms and therapeutic interventions, viable urine-derived Fabry disease cells could present a valuable tool.

## Results

### Characterization of urine-derived cells of Fabry patients

**Figure 1:**
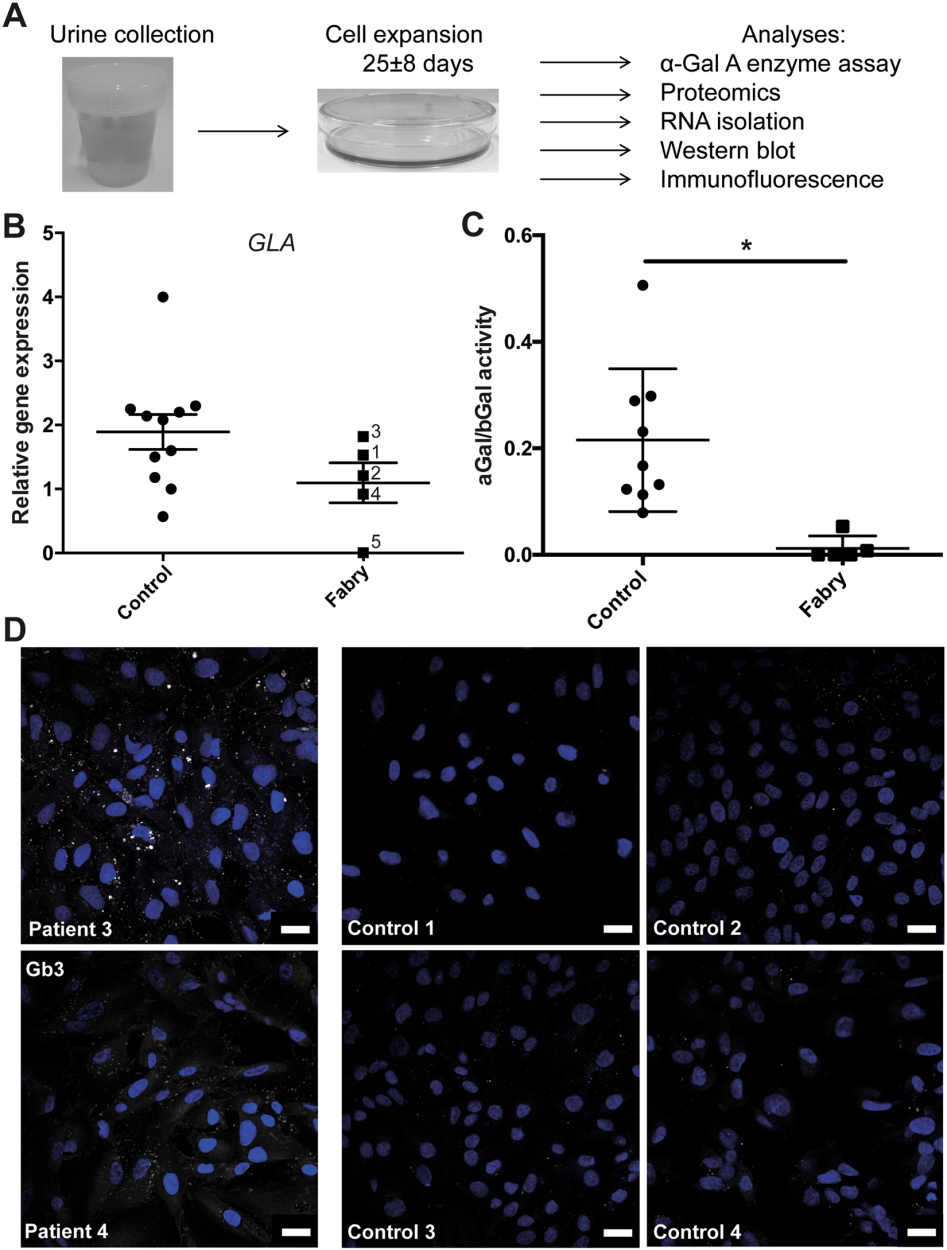
Urine-derived cells from Fabry patients. (**A**) Workflow of urine collection and primary cell culture from fresh urine. Experimental analyses described in this study are listed. (**B**) RT-qPCR of *GLA* expression, normalized against *ACTB* expression in control and Fabry patient urine-derived cells which are indicated by their patient number, (mean ± SEM, n = 5 patients and 11 controls, 2-tailed t-test, p = 0,128. (**C**) α-Gal A activity of patient samples depicts a decrease in enzyme activity compared to control samples (mean ± SEM, n = 5 patients and 9 controls, 2-tailed t-test, p = 0.0063). (**D**) Immunofluorescence of Gb3 (white), counterstained with DAPI (blue) of control and Fabry patient urine-derived cells (scale bar 20 µm).

We cultured urine-derived cells^8,11^ of 7 individuals carrying different mutations in the *GLA* gene and of gender-matched healthy individuals as controls (Fig. 1A). Patient characteristics and their clinical parameters are described in Table 1. To characterize the two cell culture subsets for their cellular composition and characteristics, we performed gene expression analysis. RT-qPCR did not reveal significantly different expression of cellular markers (Supplementary Table S1) or altered levels of α-Gal A expression (Fig. 1B, Supplementary Fig. S1A–D). In patient 5 low levels of α-Gal A expression are detected due to affection of the splice site of intron 2 resulting in increased mRNA degradation. This finding is in contrast to the male individuals carrying missense mutations (patient 3 and 4) of which α-Gal A expression is not changed. In addition, in females carrying missense or nonsense mutations (patient 1 and 2) we show that α-Gal A expression is not altered (Fig. 1B, Supplementary Fig. S1B–D). We also analyzed urine-derived cells of patients and controls for their α-Gal A activity. As expected, we detected a significant decrease of α-Gal A activity in all patient samples compared to controls. In the three samples obtained from male patients no α-Gal A activity could be detected, in contrast to residual enzyme activity measured in female patient samples (patient 1 and 2) (Supplementary Fig. S1E). Grouping patients and controls according to the ratio of α-Gal A versus ß-Gal activity (internal assay control) reveals a significant difference between patient cells and control cells (Fig. 1C). Immunofluorescence staining for Gb3 shows varying, but clear Gb3 accumulation in affected individuals’ cells (patient 3 and 4) (Fig. 1D).

**Table 1:** Patient characteristics.

Patient	Age (yr)^A^	Gender	Genetic variant	Amino acid change	Creatinine mg/dl	eGFR CKD-EPI (2009)	Albuminuria mg/g creatinine^C^	ERT^D^
1	52	F	c.427 G > A	p.A143T	0.65	102	3	alfa
2	68	F	c.679 C > T	p.R227X	0.52	100	7	alfa
3	26	M	c.124 A > G	p.M42V	1.02	101	1909	alfa
4	44	M	c.62 T > C	p.L21P	5.81	11	2013	alfa
5	61	M	c.369 + 1 G > A (IVS2 + 1 G > A)		1.38	55	2954	alfa
6	50	M	c.679 C > T	p.R227X	8.43^**B**^	7	108	beta
7	45	M	c.679 C > T	p.R227X	0.68	115	21	beta

^A^Age at day of urine collection.

^B^Patient is on peritoneal dialysis.

^C^All patients with albuminuria >30 mg/g creatinine are on ACE inhibitors.

^D^Enzyme replacement therapy (ERT) by Agalsidase alfa or beta.

**Figure 2:**
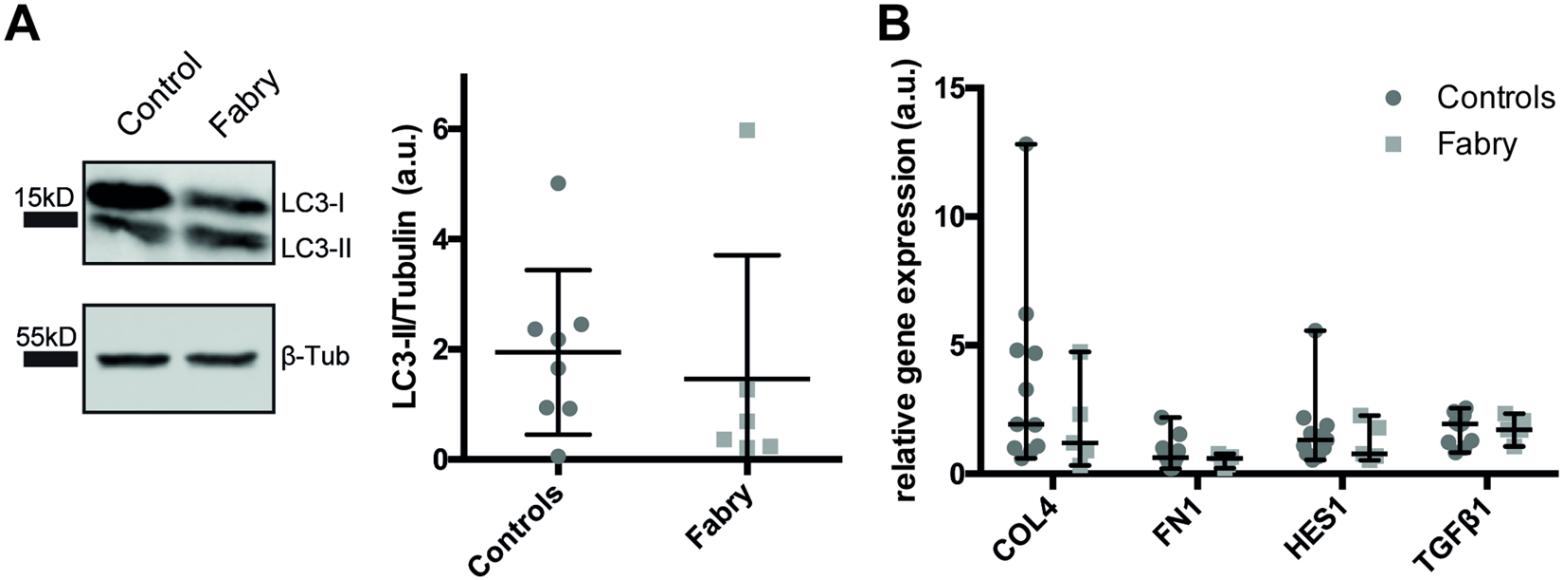
Characterization of urine-derived cells. (**A**) Representative cropped LC3-II and β-tubulin immunoblot and densitometric quantification of LC3-II immunoblot. (**B**) and intensity of LC3-II was normalized to β-tubulin band intensity of the same membrane (mean ± SEM, n = 6 patients and 8 controls, 2-tailed t-test, p = 0,6328). The molecular masses of standard proteins of the size marker are indicated. Full-length blots are presented in Supplementary Fig. S2. (**B**) RT-qPCR of *HES*, *TGFβ*, *Col*, *FN1* expression, normalized against *ACTB* expression in control and Fabry patient urine-derived cells (mean ± SEM, n = 5 patients and 11 controls, 2-Way ANOVA with Sidak multiple comparisons testing, p-values: COL4: 0,2940; FN1: 0,9965; HES1: 0,9849; TGFß1: > 0,9999).

In previous studies we and other groups detected a dysregulation of the autophagy machinery upon α-Gal A deficiency and Gb3 accumulation^12,13^. To measure if urine-derived cells exhibit dysregulated autophagy, we examined autophagy marker LC3-II protein levels by western blot (Fig. 2A, Supplementary Fig. S2). Interestingly, no significant differences between the two groups of primary cells were observed (Fig. 2A). Further studies showed *COL4*, *FN1*, *HES1*, and *TGFβ1* gene expression levels to be elevated in immortalized human podocytes in response to lyso-Gb3 *in vitro*^14^. In our primary urine-derived Fabry cells the expression of these markers was not increased (Fig. 2B).

### Label-free quantitative proteomic analysis of urine-derived cells

**Figure 3:**
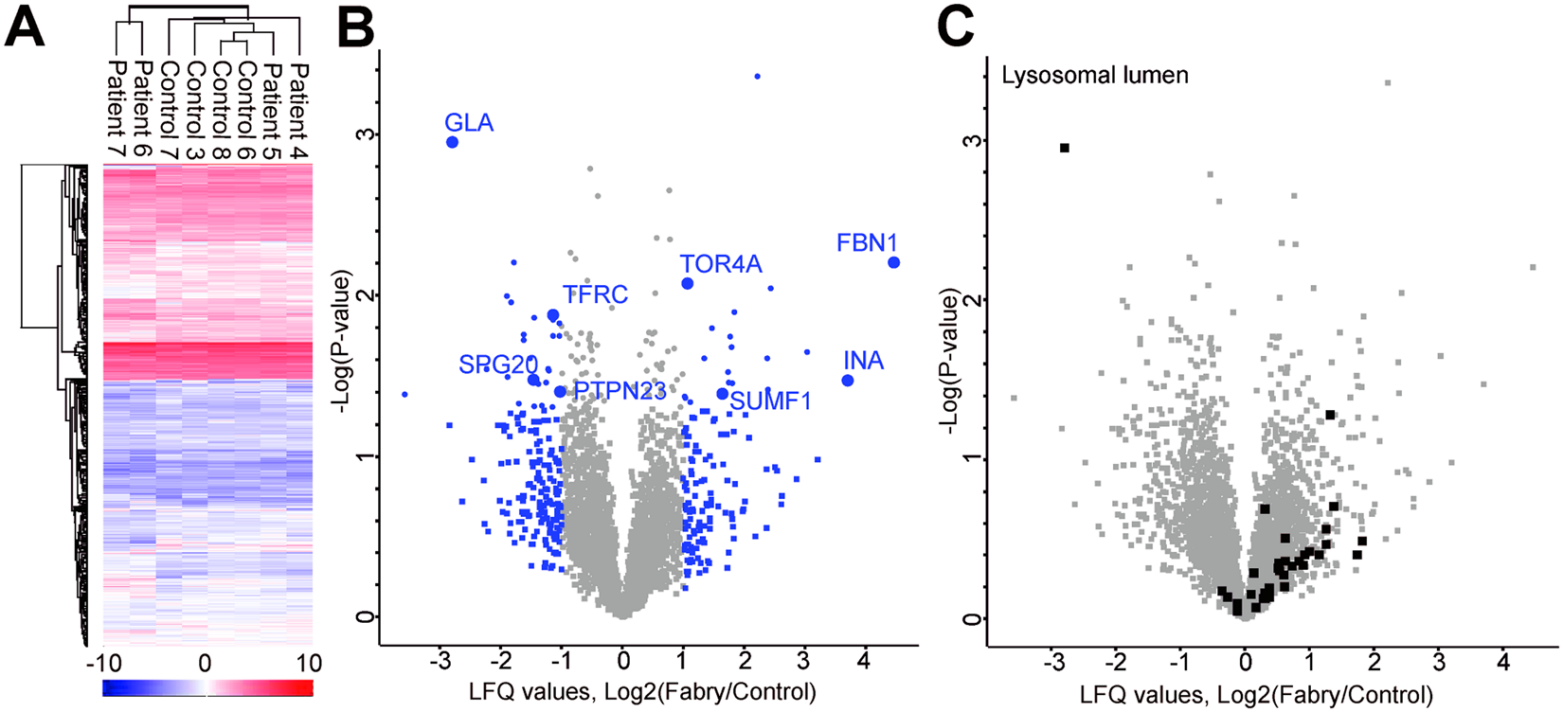
Label-free quantitative proteomic analysis by mass spectometry of urine-derived cells from Fabry patients. (**A**) Hierarchical clustering of proteins (label-free quantification (LFQ) values) within 4 Fabry patient and 4 control cell samples based on Euclidean distance. Heat map displays normalized log2 LFQ intensities of all n = 3646 proteins quantified across the samples (red = high, blue = low intensity). (**B**) Volcano plot showing logarithmized fold change of LFQ values measured from Fabry patient vs. control samples. Ratios plotted against the negative logarithmic *P* value of the Student’s *t*-test. Each dot represents a protein. α-Gal (gene *GLA*) was the most significantly and strongly decreased protein in Fabry patient cells. Proteins with a >2 fold difference (log2 ratio > 1 or < −1) are marked in blue. Proteins with a >2 fold difference and p < 0.05 are marked in blue including selected gene names. The comprehensive list is provided in Table 2. (**C**) The same Volcano plot as in B with lysosomal lumen proteins (GOCC) depicted in black.

To characterize urine-derived Fabry disease patient cells more comprehensively, we performed label-free quantitative nLC-MS/MS analysis of four male patients, including patient 4 carrying a missense mutation, patient 5 with a splice site defect, and patients 6 and 7 carrying nonsense mutations (Table 1). Patient 6 and 7 were not included in the previous qPCR and enzyme activity analysis due to limited sample size. In total, 3646 proteins were quantified (Fig. 3A). Hierarchical clustering of proteomic profiles based on Euclidean distance revealed some similarities between controls, and also strong clustering of patient 6 and 7, two siblings (Fig. 3A). Analysis of specific cellular markers on the protein level revealed no significant differences between Fabry patient-derived cells and controls (Supplementary Table S1). When analyzing the proteins being differentially expressed comparing our four urine-derived Fabry patient samples to controls, we found a strong decrease of α-Gal A expression in patient samples (Fig. 3B and Table 2, Supplementary Table S2). Since other lysosomal storage diseases have been shown to influence lysosomal proteostasis^15^, we analyzed proteins connected to lysosomal function and biogenesis. Lysosomal lumen proteins (GOCC) were increased in Fabry patient cells, thereby suggesting that urine-derived cells can be used as a suitable model for Fabry disease (Fig. 3C). Furthermore, extracellular matrix (ECM) proteins like fibrillin (FBN1) and alpha-internexin (INA) were found to be more abundant. We also observed protein alterations depicting defective endosomal trafficking (SPG20, PTPN23, TFRC) and lysosomal biology (TOR4A) (Fig. 3B).

**Table 2:** Uine-derived cells – 46 candidates with altered expression.

Sequence coverage [%]	−Log Student’s T-test p-value F_C	Student’s T-test Difference F_C	Majority protein IDs	Protein names	Gene names
19,5	3,36358806	2,2115078	A0A0A0MRZ4; Q15025; E7EMV7	TNFAIP3-interacting protein 1	TNIP1
7,5	1,64741466	3,03378963	A0A1B0GW05		
27,6	2,2037849	−1,78379869	A6NJD9; A8MVT4; A8MYK1; Q16540; H7C2P7	39S ribosomal protein L23, mitochondrial	MRPL23
14,3	1,84607609	−1,14091063	A8MUM1; Q53HC9; F8WDS8; C9J0U9; D3YTH5; F8WDX0; F8WB12	Protein TSSC1	TSSC1
35,9	1,60937079	−1,51865244	C9JQV0; Q9BRJ6; H7C0T1; H7C2R9	Uncharacterized protein C7orf50	C7orf50
9,9	1,30704371	−1,15452433	E7EU13; Q96P48; A0A0A0MSJ2; F5GWN4; F8WBT0	Arf-GAP with Rho-GAP domain, ANK repeat and PH domain-containing protein 1	ARAP1
22,7	1,38842746	1,63946486	E9PF05; Q8NBK3; F5GXA0	Sulfatase-modifying factor 1	SUMF1
7,2	1,44982613	−1,37898397	F8W9S7; Q14C86; B0QZ65; B4DGD8; C9IZ08	GTPase-activating protein and VPS9 domain-containing protein 1	GAPVD1
12,2	1,74547572	−1,139431	J3KN32; Q9UKL0	REST corepressor 1	RCOR1
16,5	1,33750558	1,11249781	J3KNF4; O14618	Copper chaperone for superoxide dismutase	CCS
44,8	1,95733784	−1,8259573	J3KRP6; J3QS72; X6R3J2; J3QQM2; J3KT74; J3QQW2; Q15532; J3QSB3; F5GWN1; J3QSG1; J3QQW6; J3QLJ7; J3QQX5; J3KT22; B4DLD3	Protein SSXT	SS18
22,5	1,31045683	−1,44714165	J3QQJ0; Q9UHR5; X6R3T8; J3QLH3; J3KRR6	SAP30-binding protein	SAP30BP
13,8	1,60731326	1,34385443	K7EL81; Q9BZL4	Protein phosphatase 1 regulatory subunit 12 C	PPP1R12C
100	1,52612046	1,7319355	K7EQX8; Q6ZR64		MXRA7
23	1,79556235	1,46848154	O60831; A6NM71	PRA1 family protein 2	PRAF2
4,2	1,31671159	−1,31916904	O94915; H0Y9X0	Protein furry homolog-like	FRYL
19,5	1,45455248	1,80712652	O95822	Malonyl-CoA decarboxylase, mitochondrial	MLYCD
50	1,87561059	−1,14349508	P02786; G3V0E5	Transferrin receptor protein 1; Transferrin receptor protein 1, serum form	TFRC
49,3	1,82593011	−1,0402894	P02787	Serotransferrin	TF
22,8	2,951003	−2,79159164	P06280; V9GYN5	Alpha-galactosidase A	GLA
64,1	1,38469508	−3,57697392	P13746	HLA class I histocompatibility antigen, A-11 alpha chain	HLA-A
37,1	2,20389005	4,45898819	P35555	Fibrillin-1	FBN1
8,6	1,41710999	2,38675785	P39880	Homeobox protein cut-like 1	CUX1
6,4	1,7555979	−1,62469578	P42858	Huntingtin	HTT
45,7	1,37415071	1,02663898	P49755; G3V2K7	Transmembrane emp24 domain-containing protein 10	TMED10
28,7	1,60610264	2,37490463	P56134	ATP synthase subunit f, mitochondrial	ATP5J2
24,5	1,46112134	1,74879551	P62745	Rho-related GTP-binding protein RhoB	RHOB
75,5	1,53432494	−1,20722389	Q06323	Proteasome activator complex subunit 1	PSME1
15,2	1,54450905	−1,21794605	Q13042; Q5T8C6	Cell division cycle protein 16 homolog	CDC16
34,2	1,74106284	1,76797581	Q13113	PDZK1-interacting protein 1	PDZK1IP1
41,6	1,32778107	−1,70735455	Q15024	Exosome complex component RRP42	EXOSC7
18,1	1,74605314	−1,03458118	Q15773	Myeloid leukemia factor 2	MLF2
56,9	1,47356668	3,69771004	Q16352	Alpha-internexin	INA
7,3	1,46114676	−1,25552082	Q5T0F9; H7C1U3; H0Y517	Coiled-coil and C2 domain-containing protein 1B	CC2D1B
3	1,72296639	−1,61854792	Q5VW36	Focadhesin	FOCAD
20	1,47918405	−1,46710634	Q8N0X7	Spartin	SPG20
12,8	1,8590069	−1,45174217	Q8NB90	Spermatogenesis-associated protein 5	SPATA5
37,5	1,89352886	1,84231853	Q8NBN7; G8JLA1	Retinol dehydrogenase 13	RDH13
10,1	1,36431703	1,03176403	Q9BVG9; E9PS47; E9PLE4	Phosphatidylserine synthase 2	PTDSS2
30,3	1,99419336	−1,89555502	Q9BYC8	39S ribosomal protein L32, mitochondrial	MRPL32
15,3	1,40200765	−1,03030014	Q9H3S7	Tyrosine-protein phosphatase non-receptor type 23	PTPN23
41,6	1,49683138	−1,88535976	Q9NRW3	DNA dC- > dU-editing enzyme APOBEC-3C	APOBEC3C
12,6	2,04344637	2,42999411	Q9NX08	COMM domain-containing protein 8	COMMD8
15,4	2,07167401	1,06417036	Q9NXH8	Torsin-4A	TOR4A
6,7	1,54149863	−2,22812366	Q9UBM7; H0YE57	7-dehydrocholesterol reductase	DHCR7
9,5	1,67767331	1,79071379	Q9Y653; H3BRH0; H3BSJ6	G-protein coupled receptor 56; GPR56 N-terminal fragment; GPR56 C-terminal fragment	GPR56; ADGRG1

### Proteome comparison of urine-derived patients cells and α-Gal A-deficient human podocytes

**Figure 4:**
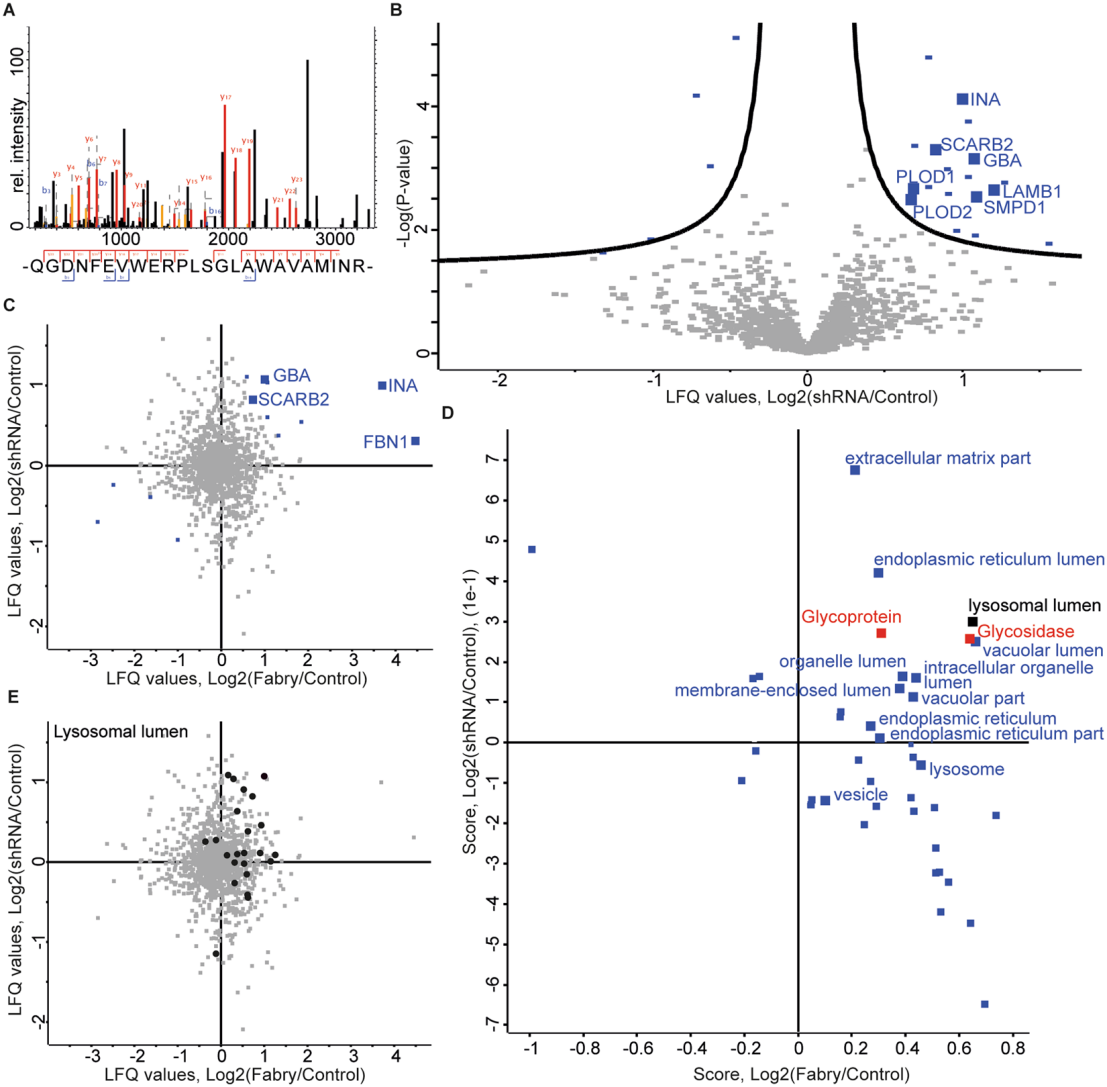
Label-free quantitative proteomic analysis in an α-Gal A-depleted human podocyte cell culture model shows an increase of lysosomal proteins in both cell culture models. (**A**) Proteome analysis of control podocytes and α-Gal A-depleted podocytes (shRNA894). MS2 spectrum of the identified *GLA* peptide (P06280, residues 333–356 QGDNFEVWERPLSGLAWAVAMINR) which was identified only in control samples but not in knockdown samples. (**B**) Volcano plot showing logarithmized fold changes of label free quantification (LFQ) values in knockdown vs. control podocyte samples. Log2 ratios of shRNA over control are plotted against the negative logarithmic *P* value of the Student’s *t*-test. Each dot represents a protein (FDR = 0.2, s0 = 0.1). Significant proteins are marked in blue with their respective gene symbol (n = 6). (**C**) Scatter plot analysis of fold changes in podocytes and urine-derived cells with proteins of interest displayed in blue. Log2 fold changes of the podocyte *GLA* knockdown model (shRNA/control) are plotted against the Log2 fold changes of the original primary urine cells (Fabry vs control). Proteins regulated positively in both datasets are marked with their respective gene symbol. (**D**) Scatterplot of 2D GO enrichment analysis in podocytes and urine-derived cells. Fold changes of both datasets were subjected to dimensionality reduction using 2D GO enrichment algorithm (FDR 0.05). Differently distributed GO terms are in blue. The GO-CC term “lysosomal lumen” is marked in black. Uniprot keywords related to the disease mechanism (Glycosidase, Glycoprotein) are labeled in red. (**E**) Mapping of lysosomal lumen proteins on initial scatter plots. Lysosomal lumen proteins are marked in black.

To investigate to what extent the observed proteome changes may be explained by the absence of α-Gal A we made use of our previously established human podocyte model of Fabry disease, which exhibits defects in lysosomal biogenesis upon shRNA-mediated knockdown of α-Gal A activity^13^. Quantitative proteomic analysis of α-Gal A knockdown podocytes and control podocytes detected α-Gal A protein in control podocytes only (Fig. 4A). In total 1387 proteins were quantified. Among proteins being increasingly expressed in α-Gal A knockdown podocytes were lysosomal proteins GBA, SCARB2, LAMB1, SMPD1, the extracellular protein INA as well as PLOD1 & PLOD2, an ECM generating protein (Fig. 4B, Table 3, Supplementary Table S3). Furthermore, we detected alterations in components and regulators of proteasomal degradation and vesicular transport (SEC23B, PSMF1, ANXA4, VPS4B, PSMB7).

**Table 3:** Human podocytes – 22 candidates with altered expression.

Sequence coverage [%]	−Log Student’s T-test p-value sh_co	Student’s T-test Difference sh_co	Majority protein IDs	Protein names	Gene names
36	2,85519877	1,0396115	A0A075B730; A0A087X1U6; P58107	Epiplakin	EPPK1
4,6	1,77468291	1,56262144	H7BYX6; A0A087WTE4; A0A087WTF6; A0A087WX77; A0A087WWD4; A0A087WV75; P13591	Neural cell adhesion molecule 1	NCAM1
34,4	2,48637642	0,67475446	E7ETU9	Procollagen-lysine,2-oxoglutarate 5-dioxygenase 2	PLOD2
9,5	3,74937353	1,04245377	E9PF17; P13611	Versican core protein	VCAN
7,8	2,64110451	1,20947615	G3XAI2; P07942; E7EPA6	Laminin subunit beta-1	LAMB1
39,9	3,14674664	1,07599513	P04062; A0A0G2JNZ5; A0A0G2JLB3;A0A0G2JNZ0	Glucosylceramidase	GBA
36,8	2,58143162	0,91128731	P07602; C9JIZ6	Prosaposin; Saposin-A; Saposin-B-Val; Saposin-B; Saposin-C; Saposin-D	PSAP
5,3	3,35784819	0,69302813	P11047	Laminin subunit gamma-1	LAMC1
9,5	2,53155615	1,09431934	P17405; G3V1E1; E9PQT3	Sphingomyelin phosphodiesterase	SMPD1
47,7	4,79110095	0,78481261	P21589	5-nucleotidase	NT5E
11,9	1,84449865	−1,01506233	P42574	Caspase-3; Caspase-3 subunit p17; Caspase-3 subunit p12	CASP3
70,2	4,17248433	−0,71968714	P63104; E7EX29; B0AZS6; E7ESK7	14-3-3 protein zeta/delta	YWHAZ
25,7	2,66470613	0,68431664	Q02809	Procollagen-lysine,2-oxoglutarate 5-dioxygenase 1	PLOD1
36,2	3,03180095	−0,6237189	Q08257; A6NP24; C9JH92	Quinone oxidoreductase	CRYZ
20,7	3,29769817	0,82711792	Q14108	Lysosome membrane protein 2	SCARB2
9,6	2,98169749	0,90587807	Q15437; Q5QPE2	Protein transport protein Sec. 23B	SEC. 23B
14	4,11857959	1,0019083	Q16352	Alpha-internexin	INA
9,3	2,76383911	1,27852472	Q5QPM9; Q5QPM7; Q92530	Proteasome inhibitor PI31 subunit	PSMF1
3,1	1,98292355	0,96811295	Q5SRE5	Nucleoporin NUP188 homolog	NUP188
4,3	1,90394767	1,08599822	Q8TEX9; H0YN14	Importin-4	IPO4
24,8	2,69318787	0,78524431	Q96HE7	ERO1-like protein alpha	ERO1L
30,5	5,10236193	−0,4577287	Q9BR76; A0A087WW53; F5H390	Coronin-1B; Coronin	CORO1B

We compared the proteomes of urine-derived patient cells with the α-Gal A-deficient podocyte cell culture model. Despite a differential baseline proteome of both cell types, proteins such as lysosomal proteins GBA, SCARB2, the ECM protein FBN1 and INA were increased in both datasets, suggesting a conserved α-Gal A-dependent mechanism in both cell culture models (Fig. 4C). To visualize distribution of shared and dissimilar molecular features, we performed 2D GO term enrichment. The 2D GO term enrichment algorithm performs dimensionality reduction on diverse dimensional datasets, thereby placing commonly regulated terms in a 2D space illustrating its global regulation across different omics datasets in a statistically controlled manner^16^. Our analysis which focusses on shared features of both datasets revealed that cellular GO terms associated with *GLA* gene function were enriched in upregulated proteins in both cell culture models, including lysosomal lumen proteins, glycoproteins, and glycosidase protein (Fig. 4D). In addition, ECM proteins were increased in both cell culture models upon α-Gal A reduction. The increased abundance of lysosomal lumen proteins was visualized also on the original scatter plot (Fig. 4E).

## Discussion

In this study, we present for the first time the potential of urine-derived cells for diagnosis and pathophysiologic evaluation in patients with Fabry disease. These cultured cells provide a model to investigate the pathomechanisms underlying Fabry disease and hold the potential of monitoring therapeutic efficacy. We show that urine-derived cells from Fabry disease patients can be used to measure α-Gal A enzyme activity, Gb3 levels, RNA levels and proteomic alterations. These cells could also be immortalized for further mechanistic studies.

Urine samples of Fabry patients have been examined in the past focusing on direct measurement of Gb3 and lyso-Gb3 levels in patients’ urine^17–19^. In this study, we investigated the cellular material derived from fresh urine samples. While urine Gb3 measurements proved to be very inconsistent^17–19^, the enzyme activity measurements we performed in urinary cells yielded stable results. This observation marks a promising step towards a non-invasive diagnosis in Fabry disease, as urinary cells could be implemented as diagnostic material via enzyme activity assays.

Investigation of renal and especially glomerular pathology of Fabry disease has been relying on immortalized human cell culture models in the past, since the *Gla* knockout mouse and the *Gla* knockout rat do not present with an overt kidney phenotype^20,21^. Patient-derived cells mark a step closer to the actual human pathology, as these cells carry the disease-causing mutation.

We show here that urine-derived cells can be used for research applications, including transcriptomic and proteomic analyses. In contrast to previous proteome studies of a single patient^22^ we compare several patient cell samples which are all carrying mutations in the *GLA* gene. Despite inter-individual variability, we identified a large number of lysosomal lumen proteins to be more abundant in patient samples compared to control samples. This finding indicates an upregulation of lysosomal proteins due to α-Gal A impairment. To our knowledge, this is the first description of an overall upregulation of lysosomal hydrolases in Fabry disease. Interestingly, the expression of most of these proteins underlies the control of the Transcription Factor EB (TFEB)^23^. Analysis of our Fabry patient dataset revealed an upregulation of known TFEB target proteins involved in lysosomal biogenesis, such as SUMF1, thus providing additional evidence for an involvement of the CLEAR network^23,24^. This network was first characterized in 2011 as a system of lysosomal and autophagosomal proteins orchestrated by the transcription factor EB controlling major cellular clearance pathways^23^. Our data suggest that TFEB is an important regulator in Fabry disease, also shown in other lysosomal storage disorders^25^. In addition, Torsin-4A (TOR4A) was enriched in Fabry patient cells. Higher Torsin-4A levels have already been shown to correlate with defective lysosomal biology in fibroblasts from patients suffering from Nieman-Pick’s disease^26^. Proteins dependent on or involved in endosomal trafficking are reduced in our patient samples. SPG20 binds with micromolar affinity to the endosomal sorting complex required for transport via (ESCRT)-III^27^. In a related manner PTPN23 facilitates endosomal sorting and multi-vesicular body (MVB) formation via ESCRT-I and TFRC as the first component of the endocytotic mechanism for iron uptake^28^. All three proteins were found to be less abundant in Fabry cells compared to healthy controls possibly indicating a defective endosomal trafficking pathway. Lysosomal lumen proteins are consistently increased in both α-Gal A-defective podocytes and urine-derived α-Gal A-deficient cells. In addition, extracellular matrix (ECM) proteins are increased in both α-Gal A*-*deficient podocytes and urine-derived cells from patients. The ECM proteins fibrillin and alpha-internexin were also more abundant in patient samples, eluding to the finding that interstitial fibrosis and scarring are often observed in kidney biopsies of Fabry patients^29,30^. These markers have the potential of searching for and monitoring renal fibrosis in Fabry patients or to accompany renal biopsies in Fabry patients to yield further insights into disease pathology^2,31^.

Overall, we demonstrate here that primary urine-derived cells reflect some, but not all, of the properties of Fabry disease. Some of the differences observed between primary urinary cells and known cell culture models may be explained by tissue-specific stress responses. In contrast to immortalized human podocytes exposed to lyso-Gb3, expression levels of *COL4*, *FN1*, *HES1*, and *TGFβ1* were not elevated in urine-derived cells of Fabry disease patients^14^. Likewise, we did not find evidence for alterations of autophagy in these cells, as we reported earlier in a study of α-Gal A-deficient human podocytes^13^, and Chévrier *et al*. reported in human lymphoblasts and fibroblasts^12^. These variations may occur due to different cell types and different cell culture conditions used.

We have analyzed a limited number of patient samples in our study. Due to the high clinical variability between different individuals it is critical to confirm our results in a larger cohort of Fabry patients. This study may be a starting point and can direct us to candidate proteins and pathways to investigate and to validate in follow-up proteome studies of urine-derived cells.

In conclusion, urine-derived cells are an effective source of primary patient material for future studies of pathomechanisms in Fabry disease. They also hold the potential of monitoring Fabry patient therapy. The proteomic data we obtained from patient-derived urinary cells could be used as a first step in evaluating prognostic markers detectable in patient urine. Besides using these cells as diagnostic tools, testing of therapeutic interventions is feasible. In the context of personalized medicine, primary urine-derived cells could be exposed to different types of compounds or even gene-editing strategies to test their therapeutic capacity.

## Methods

### Primary urine cell culture

Urine-derived cells were obtained from Fabry patients and healthy gender-matched controls as previously described^11,22^. Cells isolated from fresh urine (40–125 ml) were plated in a non-coated 24-well plate, and cells were expanded up to passage 3. The glucose concentration in primary medium is 3.15 g/L, and in proliferation medium 2.9 g/L. Since we preferred to keep the passage of the primary cell culture at maximum three, not all experiments were performed with cells of all seven affected individuals. Cells were not immortalized.

### Human podocyte cell culture

The conditional immortalized human podocytes, control podocytes and podocytes with reduced α-Gal activity were cultured as previously described^13^. Briefly, immortalized podocytes were transduced with co-shRNA (control), and with shRNA 894 (α-galactosidase A knockdown) and cultured in RPMI media (Sigma-Aldrich, Taufkirchen, Germany) with insulin-transferrin-sodium selenite as supplement (ThermoFisher Scientific, Waltham, USA) containing 10% fetal bovine serum (Biochrom, Berlin, Germany). Cells proliferated at 33 °C until they reached a confluence of 60–70%. Afterwards cells were differentiated at 37 °C for 14 days before harvesting them for proteomic analysis. Cells were regularly tested for mycoplasma infection using mycoplasma detection kit from Minerva biolabs (Minerva Biolabs, Berlin, Germany).

### Immunofluorescence

Urine-derived cells (passage 1 or 2) were grown on coverslips to a confluency of 80%. After fixation with 4% PFA and blocking with normal donkey serum in PBS containing 0.1% triton-X, coverslips were incubated with rat anti-Gb3 (CD77) (Abcam, ab19795) o/n at 4 °C. Secondary antibody staining was performed for 1 hour at room temperature with Cy3-conjugated goat anti-rat IgM µ chain specific (Jackson, 112-165-020). Coverslips were mounted in ProLong Diamond with DAPI (ThermoFischer, P36962) and imaged with a ZEISS LSM710 confocal microscope (Zen software).

### Nano-liquid-chromatography-(nLC)-MS/MS proteomic analysis

All urine-derived cell samples were harvested at passage 3 from a 70% confluent 100 mm petri dish. PBS-washed cell pellets were snap-frozen in liquid nitrogen and stored at −80 °C until further processing. Podocytes and urine-derived cell samples were prepared as described previously^22^. In short, cells were lysed in 8 M urea, 50 mM ammonium bicarbonate and protease inhibitors. After centrifugation at 4 °C, protein concentrations were measured with the Pierce BCA protein assay kit (Thermo Scientific). Protein lysates were reduced using 10 mM DTT, followed by 40 mM iodacetamide reduction, both 45 minutes at RT in the dark. Finally, 20 µg of proteins were digested using trypsin at RT in the dark. Overnight digestion was stopped by adding 0.5% formic acid, and peptides were cleaned and desalted with stop-and-go extraction tips (Stagetips)^32^. Before MS/MS analysis, peptides were resuspended in 0.1%FA and nLC fractionation of peptides was performed using a 1 hour (cultured human podocytes) or 2,5 hour (urine-derived cells) gradient with a binary buffer system as previously described^33^. Peptides were analysed using a quadrupole-orbitrap based QExactive Plus mass spectrometer (Thermo Scientific)^34^.

### Statistics

Raw files were quantified and normalized using the MaxQuant version 1.5.5.1^35^ with default settings and using the LFQ algorithm^36^. Match between runs option was enabled. The human reference proteome without isoforms was used as a database (downloaded from Uniprot December 2016). Peptide, PSM and protein FDR were 0.01. MaxQuant output (protein group files) were analysed using Perseus 1.5.5.3^16^. For the primary urinary cell data, search results containing reverse hits were removed, as well as contaminants and proteins identified by site only. LFQ expression values were logarithmized (log2). Imputation of the missing values was performed when four values were present in each group with default parameters (downshift 1.8 SD, width = 0.3). Normalization was performed by subtracting the median from all samples. In all cases, proteins were annotated with GO terms, Pfam (protein families) domains and kyoto encyclopedia of genes using the Perseus main annotations file (April 2015). Statistical overrepresentation of the respective categories was performed using Fisher’s exact test (using a cut-off of *P* = 0.05) and at least three proteins within a category. Volcano plots were generated by plotting the negative logarithmized p value (−log10) vs the log2 ratio of expression values in Fabry patients vs controls. Differentially changed proteins were determined by a p-value < 0.05 (−logp > 1.301) and a log2 fold-change of bigger than 1 or smaller than −1. For the cultured human podocyte data, we accepted a number of 8/12 valid values. Data were logarithmized, and processed as described above. To determine two-tailed t-test was performed using a method similar to SAM^37^ with FDR = 0.2 and s0 = 0.1 and a two-tailed t-test. 2D GO enrichment^16^ was used for comparison of both datasets after matching the Uniprot data from the cultured podocytes on the urine-derived cells quantification. Log2 fold changes were used as input for the algorithm as previously described^38^, and differentially distributed GO terms were plotted in a scatter plot after FDR correction FDR < 0.05.

### Raw data

The raw data were deposited in the PRIDE proteomExchange repository. Primary cultured urinary cells from Fabry disease patients: Project accession: PXD007081, Username: reviewer25073@ebi.ac.uk, Password: wXEJOQ0H. Cultured podocytes with alpha-Galactosidase knockdown: Project accession: PXD007080, Username: reviewer83301@ebi.ac.uk, Password: 9cPQdp1U.

### RT-qPCR

Cells were lysed and total RNA was isolated (TRI Reagent, Sigma Aldrich). RNA concentration was measured (Nanodrop 1000 spectrophotometer, Peqlab) and cDNA was synthesized from 1000 ng RNA template using the High Capacity cDNA RT Kit (Applied Biosystems) according to the supplier’s protocol. Dilutions were made for RT-qPCR analysis to determine mRNA expression levels which were normalized against a reference gene. The Power SYBR Green PCR Master Mix (Applied Biosystems) was used to multiply and measure the cDNA with a 7900HT Fast Real-time PCR System (Applied Biosystems). The following PCR program was used: 95 °C for 10 min, followed by 40 cycles of 15 s at 95 °C, 60 s at 60 °C, then 15 s at 95 °C followed by a melt of the product from 60 °C-95 °C. The primer sequences (IDT) used are: hACTB forward 5′-GGACTTCGAGCAAGAGATGG, hACTB reverse 5′-AGCACTGTGTTGGCGTACAG, hHPRT1 forward 5′-TGACACTGGCAAAACAATGCA, hHPRT1 reverse 5′-GGTCCTTTTCACCAGCAAGCT, Patient 5 mutation spanning hGLA forward 5*′-*TGGAAGGATGCAGGTTATGAG, hGLA reverse 5*′-*CCCTAGCTTCAGTCCTTTGCT, Patient 3 & 4 mutation spanning hGLA forward 5*′-*CGCGCTTGCGCTTCG, hGLA reverse 5*′-*CCAACAGTCATCAATGCAGAGG, Patient 1 mutation spanning hGLA forward 5*′-*GCAAAGGACTGAAGCTAGGGA, hGLA reverse 5*′-*GGAGTACACAATGCTTCTGCC, Patient 2 & 6 & 7 mutation spanning hGLA forward 5*′-* GCCCTTTCAAAAGCCCAATTAT, hGLA reverse 5*′-*AGGGCCATCTGAGTTACTTGC, hHES1 forward 5′-ACCAAAGACAGCATCTGAGCA, hHES1 reverse 5′-GCCGCGAGCTATCTTTCTTCA, hCOL4 forward 5*′-*CTCCTGGTTCTCCACAGTCAG, hCOL4 reverse 5*′-*AAGACCCTGCCAGACCAAGG, hFN1 forward 5′-GGTCCGGGACTCAATCCAAAT, hFN1 reverse 5′-ACCCTGAACTGTAAGGGTTCTT, hTGFβ1 forward 5′-CCCAGCATCTGCAAAGCTC, hTGFβ1 reverse 5′-GTCAATGTACAGCTGCCGCA. Urine cell characterization primer sequences are listed in Supplemental Table S1. The ΔΔCT method was used for statistical analysis to determine relative gene expression levels.

### Enzyme assay

Cultured primary urinary cells were collected in cold PBS. Cell lysis was performed by freezing and thawing over five cycles. Enzyme activity for α-Galactosidase A (α-Gal) and β-Galactosidase (β-Gal) was measured after incubation of lysed cells with 4-methylumbelliferyl a-D-galactopyranoside for α-Gal or 4-methylumbelliferyl b-D-galactopyranoside for β-Gal (Sigma, Taufkirchen, Germany) as described previously^39^ α-Gal A activity was normalized to β-Gal activity, functioning as an internal control.

### Western blot

We performed Western blot analysis using standard techniques. SDS-PAGE was used to resolve proteins by size while visualization was done using infrared fluorescence secondary antibodies and scanning with the LI-COR odyssey system (LI-COR Biotechnology, Bad Homburg, Germany) as described previously^40^. A molecular size marker (PageRuler, Thermo Scientific, 26620) was included. We quantified bands with the Image studio software by LI-COR. Anti LC-3 antibody (MBL, Woburn, USA) was diluted 1:500, anti beta-tubulin antibody (Developmental Studies Hybridoma Bank, University of Iowa, USA) 1:200.

### Ethics approval

Primary urine cells were derived from Fabry patients with known mutations in the *GLA* gene. The ethics committee of the University Hospital Cologne approved the study, and written informed consent was obtained from all study participants prior to entering the study. All experiments were performed in accordance with the guidelines and regulations. This study is registered at the German Registry for Clinical Studies Drks.de as DRKS00010534.

## Electronic supplementary material

Supplementary Information

Supplementary Table S1

Supplementary Table S2

Supplementary Table S3
