# Supplementary material for: Urine-derived cells: a promising diagnostic tool in Fabry disease patients
Source: Sci Rep. 2018 Jul 23;8:11042. doi: 10.1038/s41598-018-29240-w (PMC6056427; doi:10.1038/s41598-018-29240-w)
Supplement: Supplementary file 1 — Supplementary Information [file 41598_2018_29240_MOESM1_ESM.pdf]

SUPPLEMENTARY INFORMATION

Urine-derived cells: a novel non-invasive diagnostic tool in Fabry disease patients

Gisela G. Slaats[1,2,3#], Fabian Braun[1,2,3,4#], Martin Hoehne[1,2,3,5], Laura E. Frech[1,2,3], Linda Blomberg[1,2,3], Thomas Benzing[1,2,3,5], Bernhard Schermer[1,2,3,5], Markus M. Rinschen[1,2,3], Christine E. Kurschat[1,2,3*]

[1]Department II of Internal Medicine Medicine and Center for Rare Diseases Cologne, University Hospital of Cologne, Cologne, Germany

[2]Cologne Excellence Cluster on Cellular Stress Responses in Ageing-Associated Diseases (CECAD), University of Cologne, Cologne, Germany

[3]Center for Molecular Medicine Cologne, University of Cologne, Cologne, Germany

[4]III. Department of Medicine, University Medical Center Hamburg-Eppendorf, Hamburg, Germany

[5]Systems Biology of Aging, University of Cologne, Cologne, Germany

[#]These authors contributed equally

[*]Corresponding author

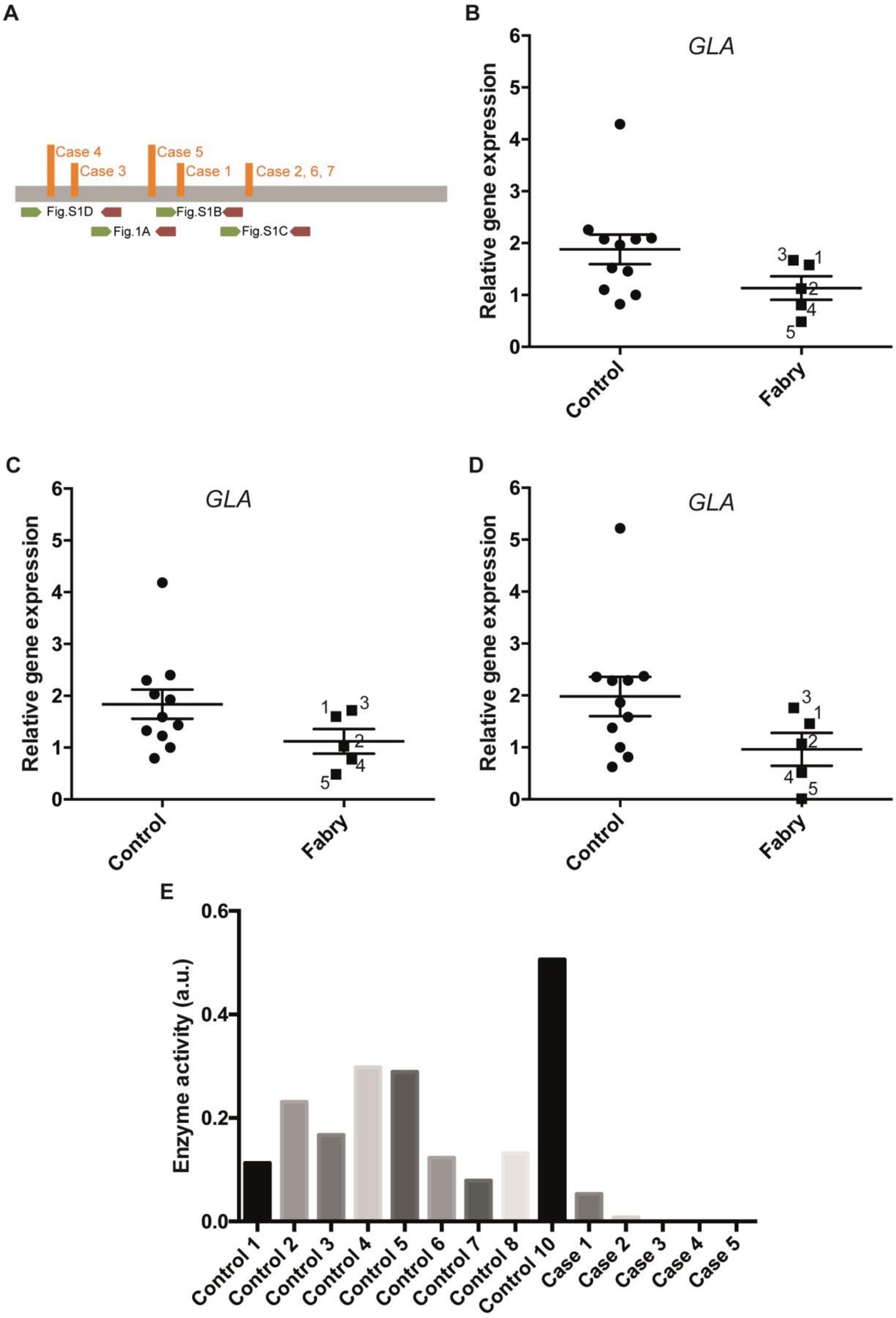
A
Case 4
Case 3
Case 5
Case 1
Case 2, 6, 7
Fig.S1D
Fig.S1B
Fig.1A
Fig.S1C
B
GLA
Relative gene expression
Control
Fabry
C
GLA
Relative gene expression
Control
Fabry
D
GLA
Relative gene expression
Control
Fabry
E
Enzyme activity (a.u.)
Control 1
Control 2
Control 3
Control 4
Control 5
Control 6
Control 7
Control 8
Control 10
Case 1
Case 2
Case 3
Case 4
Case 5

Supplemental Figure S1. Urine-derived cells from Fabry patients. (A) Schematic overview over RT-qPCR conditions. Mutations of individual cases are depicted in orange on consensus coding sequence of *GLA* gene (grey). Green arrows indicate forward primers, red arrows indicate reverse primers. Corresponding figure panels depicting the results are enclosed between forward and reverse primer. (B-D) RT-qPCR of *GLA* expression, normalized against *ACTB* expression in control and Fabry patient urine-derived cells which are indicated with their patient number, (mean ± SEM, n=5 patients and 11 controls, 2-tailed t-test, A: p=0,1226; B: p=0,1353; C: p=0,1152). Different primer pairs cover different amplicons of *GLA*, A: genetic variant: c.427 G>A; B: genetic variant: c.679C>T; C: c.124A>G. (E) α-Gal A enzyme activity. Single sample results show a wider range of enzyme activity in control samples. Residual α-Gal A activity can only be detected in cells from female patients (patients 1 & 2). Patients 3, 4 and 5 show no residual α-Gal A activity. α-Gal A activity measurements were not available for patients 6 and 7.

A

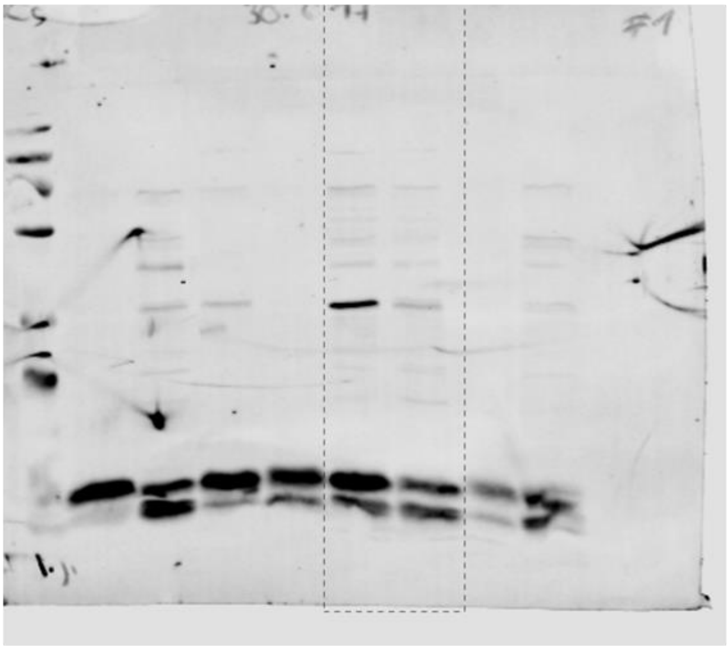

B

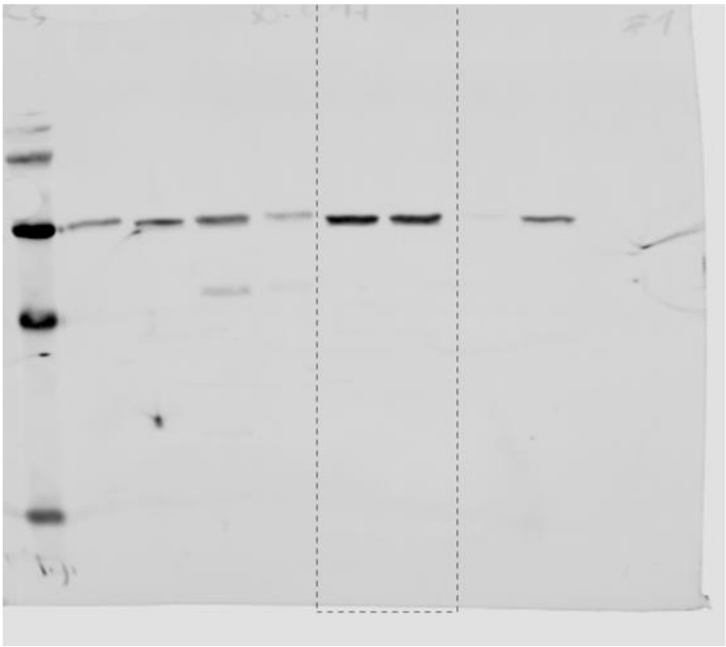

Supplemental Figure S2. Full-length immunoblots. (A) Uncropped unedited anti-LC3-II incubated membrane. (B) The same membrane as displayed in panel (A) showing the uncropped unedited β-tubulin antibody incubation of the membrane. The molecular size marker (PageRuler, Thermo Scientific, 26620) in the first lane indicates the molecular mass of standard proteins.
